# ASO Author Reflections: Beyond Time to Diagnosis: The Lasting Impact of Diagnostic Experience in GIST

**DOI:** 10.1245/s10434-026-19889-3

**Published:** 2026-06-07

**Authors:** Tessa van Amerongen, Winette T. A. Van der Graaf, Olga Husson

**Affiliations:** 1 Department of Medical Oncology, Antoni van Leeuwenhoek Hospital - Netherlands Cancer Institute, Amsterdam, The Netherlands; 2 Department of Medical Oncology, Erasmus MC Cancer Institute, Erasmus University Medical Center, Rotterdam, The Netherlands; 3 Department of Surgical Oncology, Erasmus MC Cancer Institute, Erasmus University Medical Center, Rotterdam, The Netherlands; 4 Department of Public Health, Erasmus University Medical Center, Rotterdam, The Netherlands

Efforts to improve cancer diagnosis have long focused on shortening time intervals, assuming that earlier diagnosis is inherently better.^1^ However, in rare tumors, such as gastrointestinal stromal tumors (GIST), where symptoms are often nonspecific and expertise is concentrated, diagnostic pathways are complex and poorly characterized.^2^ Data on the length and impact of diagnostic intervals in GIST remain limited. Previous work from our group demonstrated increased general practitioner (GP) consultations starting 4 months before diagnosis, suggesting diagnostic uncertainty in the pre-diagnostic phase.^3^ However, the impact of the diagnostic trajectory on patient outcomes remains insufficiently understood.

In this nationwide study of diagnostic pathways in GIST, we found that nearly half of patients experienced diagnostic intervals of ≥ 1 month, and one in five ≥ 3 months.^4^ Prolonged intervals were more likely among women, patients with small-intestinal tumors, and those with mid-sized tumors (7–12 cm). Importantly, interval duration itself was not associated with health-related quality of life (HRQoL). By contrast, patients who perceived their diagnostic trajectory as negatively impacting their well-being reported significantly worse outcomes across multiple HRQoL domains, even several years after diagnosis (mean 5.9 years). Qualitative findings supported these results, with patients describing prolonged uncertainty, frustration, and fear, as well as ongoing physical limitations they attributed to delayed care. Some patients also perceived their advanced disease to be a consequence of a prolonged diagnostic interval, reflecting the lasting psychological impact the diagnostic trajectory may have on patients’ understanding of their illness. Many attributed these experiences to healthcare-related factors, contributing to a sense of not being heard or supported. Together, these findings suggest that the experience of the diagnostic process may be more closely associated with long-term well-being than its absolute duration of the interval itself.

These observations challenge the current emphasis on time-based metrics alone and highlight the need for a more patient-centered definition of diagnostic quality. Improving timeliness remains important but should be complemented by clear communication about diagnostic uncertainty, realistic discussion of the currently uncertain relationship between diagnostic intervals and oncologic outcomes, and psychosocial support during the diagnostic phase. Future research should build on these findings through prospective data collection, for example by capturing patient and general practitioner perspectives on symptoms, concerns, and diagnostic intervals at the time of diagnosis, and by defining clinically meaningful thresholds for delay. Whether prolonged diagnostic intervals influence survival outcomes in GIST remains unclear and is currently being evaluated in ongoing analyses. As survival improves with tyrosine kinase inhibitors (TKIs), increasing numbers of patients with advanced or metastatic GIST live with long-term survivorship challenges, making it increasingly important to understand how patients experience the diagnostic trajectory and survivorship over time.^5^ Ongoing work, including our observational study on survivorship challenges in patients with advanced and metastatic GIST treated with TKIs (registered at clinicaltrials.gov: NCT07522762), aims to further address this gap by integrating clinical and patient-reported outcomes over time. Ultimately, embedding the patient’s perspective—from the first symptom to long term survivorship—is essential to improving outcomes in a disease where uncertainty is often unavoidable, but where patients should still feel heard, informed, and supported throughout the process.
